# Association between decreased ipsilateral renal function and aggressive behavior in renal cell carcinoma

**DOI:** 10.1186/s12885-022-10268-1

**Published:** 2022-11-07

**Authors:** Jang Hee Han, Seung-hwan Jeong, Sanghun Han, Hyeong Dong Yuk, Ja Hyeon Ku, Cheol Kwak, Hyeon Hoe Kim, Chang Wook Jeong

**Affiliations:** 1 Department of Urology, Seoul National University Hospital, 101 Daehak-Ro, Jongno-Gu, Seoul, 03080 Korea; 2 Department of Urology, Seoul National University College of Medicine, Seoul, South Korea

**Keywords:** Aggressiveness, Disease-free survival, Prognosis, Renal cell carcinoma, Tc 99 m-DTPA

## Abstract

**Background:**

To assess prognostic value of pre-operative ipsilateral split renal function (SRF) on disease-free survival (DFS) and its association with aggressive pathological features in renal cell carcinoma (RCC) patients.

**Methods:**

We examined patients registered in SNUG-RCC-Nx who underwent partial or radical nephrectomy at Seoul National University Hospital between January 1, 2010 and December 31, 2020. Patients with the following criteria were excluded from the study. 1) non-kidney origin cancer or benign renal tumor, 2) no pre-operative Tc 99 m-DTPA renal scan, 3) single kidney status or previous partial or radical nephrectomy, and 4) bilateral renal mass. Finally, 1,078 patients were included.

**Results:**

Among 1,078 patients, 899 (83.4%) showed maintained ipsilateral SRF on DTPA renal scan; 179 patients (16.6%) showed decreased SRF. The decreased SRF group showed significantly large tumor size (maintained vs. decreased SRF; 3.31 ± 2.15 vs. 6.85 ± 3.25, *p* < 0.001), high Fuhrman grade (grade 3–4) (41.7% vs. 55.6%, *p* < 0.001), and high T stage (T stage 3–4) (9.0% vs. 20.1%, *p* < 0.001). Pathological invasive features, including invasion of the renal capsule, perirenal fat, renal sinus fat, vein, and collecting duct system, were associated with low SRF of the ipsilateral kidney. Univariate Cox regression analysis identified higher SSIGN (The stage, size, grade, and necrosis) score and decreased ipsilateral SRF as significant risk factors, while multivariate analysis showed SSIGN (5–7) (hazard ratio [HR] 11.9, *p* < 0.001) and SSIGN (8–10) (HR 69.2, *p* < 0.001) were significantly associated with shortened DFS, while decreased ipsilateral SRF (HR 1.75, *p* = 0.065) showed borderline significance. Kaplan–Meier analysis showed that decreased ipsilateral SRF (< 45%) group had shorter DFS than the other group (median DFS: 90.3 months vs. not reached, *p* < 0.001).

**Conclusions:**

Among unilateral RCC patients, those with low ipsilateral SRF showed poor prognosis with pathologically invasive features. Our novel approach may facilitate risk stratification in RCC patients, helping formulate a treatment strategy.

**Supplementary Information:**

The online version contains supplementary material available at 10.1186/s12885-022-10268-1.

## Background

Renal cell carcinoma (RCC) incidence is increasing [1], but a clinical hurdle regarding its treatment exists owing to its heterogeneous clinical characteristics, leading to variable patient survival outcomes [2]. Although TNM stage and Fuhrman nuclear grade are well-known strong independent prognostic factors after RCC surgical treatment [3, 4], current classifications cannot perfectly stratify risk levels [2]. Accordingly, risk factors have been investigated using radiologic assessment, such as ill-defined infiltrative growth pattern [5] or absence of pseudocapsule evaluated through multidetector computed tomography (MDCT) [2] however, preoperative diagnostic accuracy is limited [5].

Recently, focusing not only the tumor itself but also on the kidney tumor-parenchyma interface has been in the spotlight. This interface is recognized as the interacting point of the immune cells and tumor, thus can be used in potential treatment strategies [6]. On the other hand, this interface is where tumor-adjacent normal parenchyma is affected by tumor expansion, which generally negatively affects organ function [7]. As renal cell carcinoma (RCC) grows, the renal parenchyma adjacent to the tumor undergoes long-standing mechanical compression from the tumor, resulting in glomerular damage and interstitial fibrosis [7]. Therefore, a high degree of compression or direct invasive/infiltrative growth may aggravate organ injury, ultimately leading to decreased ipsilateral renal function. This may be triggered by pathological characteristics, including a thin pseudocapsule, which cannot buffer tumor pressure [2] a pathologically infiltrative growth pattern [8], which is now acknowledged as an aggressive feature [9] or pseudocapsular invasion [3].

We hypothesized that aggressive RCC may induce significant parenchymal damage, leading to reduced ipsilateral renal function, as observed under the pre-operative Tc 99 m-DTPA renal scan. We assessed the prognostic value of pre-operative ipsilateral renal function, which affects disease-free survival (DFS), and its association with aggressive pathologic features.

## Methods

### Ethics approval and informed consents

This study was approved by the Institutional Review Board (IRB) of the Seoul National University Hospital (IRB no. 2205–096-1325). The requirement for informed consent was waived by Institutional Review Board (IRB) of the Seoul National University Hospital Ethic committee owing to the study’s retrospective nature. The study was performed in accordance with applicable laws and regulations, good clinical practice, and ethical principles, as described in the Declaration of Helsinki.

### Patient population and measurements

We reviewed patients registered in a prospectively collected retrospective database, SNUG-RCC-Nx, who underwent partial or radical nephrectomy at Seoul National University Hospital between January 1, 2010 and Dec 31, 2020. During this period, 4813 patients were registered in our database. Among them, Patients with the following criteria were excluded from the study: 1) patients diagnosed with non-kidney origin cancer or benign renal tumor, 2) those who did not undergo diethylenetriamine pentaacetic acid (DTPA) renal scan preoperatively, 3) those with a single kidney status or who underwent previous partial/radical nephrectomy, and 4) those with bilateral renal mass. Finally, 1,078 patients were eligible for analysis. The primary and secondary endpoints were the effect of DTPA-based split renal function (SRF) on DFS and its association with pathological findings, respectively.

### Collected parameters

**Fig. 1:**
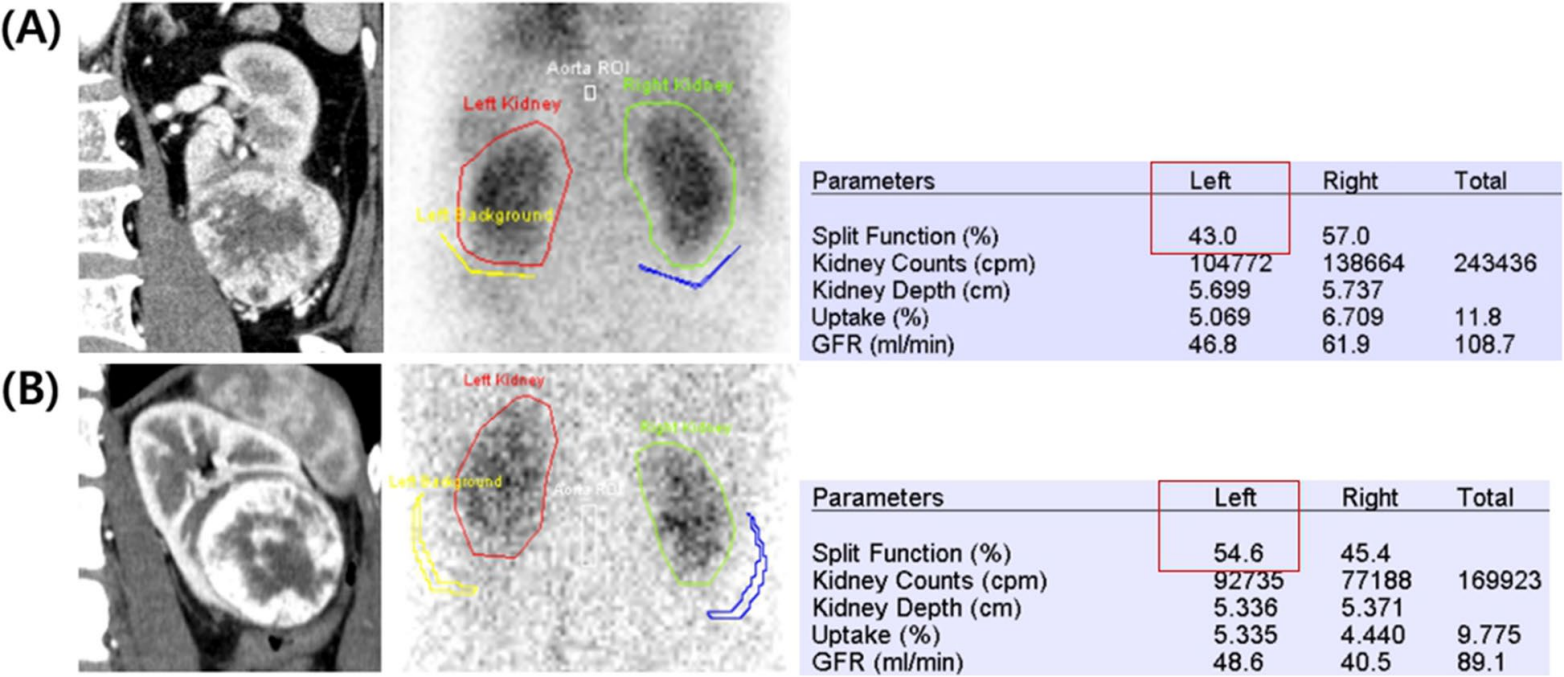
The representative image of preoperative abdominopelvic computed tomography (APCT) (left) and diethylenetriamine pentaacetic acid (DTPA) renal scan (middle) and its result (right). Patient with decreased (**A**) or maintained (**B**) ipsilateral renal function

The demographic and clinicopathological data of patients, including their sex, age at surgery, accompanying comorbidities, pre-operative imaging findings, pre-operative laboratory findings (serum creatinine, estimated glomerular filtration rate [eGFR]), pathology (size, histology, grade, and stage), DTPA findings (SRF and eGFR), and disease status (DFS) were collected. Figure 1 shows the pre-operative abdomino-pelvic computed tomography (APCT) and DTPA imaging findings in two patients. The stage, size, grade, and necrosis (SSIGN) score [10] was calculated which is the well-validated prognostic factor composed of several pathological factors. For the survival analysis, patients were stratified by collapsing scores into three categories, consisting of scores 0–4, 5–7, and ≥ 8, respectively as in the previous paper [11].

### Measurement and definition of decreased ipsilateral renal function

Tc 99 m-DTPA renal scan was performed preoperatively. Regarding laterality, the kidney affected by RCC was considered the affected side, whereas the contralateral kidney was considered the non-affected side. The region of interest was defined and SRF of the affected versus unaffected side measured. Patients with ipsilateral SRF < 45% were classified into the lowered ipsilateral renal function group, according to previous studies (normal range: 45–55%) [12, 13].

### Statistical analysis

Differences in the patient clinical and pathological characteristics in the maintained and decreased ipsilateral SRF groups were compared using independent Student’s t-test and chi-square test. Univariate and multivariate Cox regression analyses were performed to assess the independent influence of the possible risk factors on DFS. All statistical analyses were performed using SPSS version 25 software (SPSS, version 25.0.0.2, IBM Corp., Armonk, NY, USA) and R (R version 3.6.3, R Foundation, Vienna, Austria). *P* value < 0.05 was considered statistically significant; all statistical tests were two-sided.

## Results

### Patient characteristic comparison between maintained and decreased ipsilateral SRF

Among 1,078 patients, 899 (83.4%) showed maintained ipsilateral SRF on DTPA renal scan, while 179 (16.6%) showed decreased SRF. When comparing the two groups, the decreased SRF group showed significantly large tumor size (maintained SRF vs. decreased SRF; 3.31 ± 2.15 vs. 6.85 ± 3.25, *p* < 0.001), high Fuhrman grade (grade 3–4) (41.7% vs. 55.6%, *p* < 0.001), and high T stage (T stage 3–4) (9.0% vs. 20.1%, *p* < 0.001). There were no differences in age, sex, comorbidities, renal function, or histological type (Table 1).

**Table 1:** Baseline characteristics

	Maintained ipsilateral SRF (*N* = 899)	Decreased ipsilateral SRF (*N* = 179)	*p*-value
Age (years)	60.5 ± 12.7	61.5 ± 13.3	0.363
Sex (n, %)			0.531
Male	608 (68.0)	126 (70.4)	
Female	286 (32.0)	53 (29.6)	
HTN (n, %)	458 (51.1)	90 (50.3)	0.838
DM (n, %)	193 (21.5)	38 (21.2)	0.926
Dyslipidemia (n, %)	191 (21.2)	35 (19.6)	0.673
Liver disease	97 (10.8)	20 (11.2)	0.992
Tuberculosis	40 (4.4)	8 (4.5)	1.000
BMI (kg/m^2^)	25.4 ± 3.54	25.2 ± 3.64	0.636
Preoperative lab			
Creatinine (mg/dL)	1.00 ± 2.72	1.02 ± 0.78	0.942
eGFR (mL/min/1.73 m^2^)	84.8 ± 22.5	81.3 ± 24.3	0.073
Operative method (n, %)			< 0.001
Partial nephrectomy	781 (87.2)	133(74.3)	
Radical nephrectomy	78 (12.8)	38 (25.7)	
Tumor size (cm)	3.31 ± 2.15	6.85 ± 3.25	< 0.001
T stage (n, %)			< 0.001
T1–T2	815 (91.0)	143 (79.9)	
T3–T4	91 (9.0)	36 (20.1)	
Fuhrman grade (n, %)			0.001
Grade 1–2	521 (58.3)	79 (44.4)	
Grade 3–4	372 (41.7)	99 (55.6)	
Pathology (n, %)			0.357
Clear cell	756 (83.9)	145 (81.0)	
Non-clear cell	143 (16.1)	34 (19.0)	
Ipsilateral GFR (ml/min)	44.0 ± 13.8	34.6 ± 13.4	< 0.001

*SRF* split renal function, *BMI* body mass index, *eGFR* estimated glomerular filtration rate, *HTN* hypertension, *DM* diabetes mellitus

In the meanwhile, 25 patients (2.3%) showed synchronous metastasis in this cohort. Among them, 11 showed maintained ipsilateral SRF, while 14 showed decreased SRF (Supplementary Table 1). Decreased SRF group in synchronous metastasis subgroup showed significantly higher Fuhrman grade, and higher incidence of sarcomatoid component, necrosis, and renal sinus invasion. In terms of International Metastatic RCC Database Consortium (IMDC) risk group classification, all of the maintained SRF group harbored intermediate risk, while decreased SRF group showed about 20% of patients classified as high risk, and the others of intermediate risk.

### Association of decreased ipsilateral renal function with pathologic findings

A univariate logistic regression analysis was performed to assess whether aggressive pathological features were associated with decreased ipsilateral renal function. Table 2 demonstrates that renal capsule invasion (odds ratio [OR]: 1.53, *p* = 0.01), perirenal fat invasion (OR: 1.83, *p* = 0.03), renal sinus fat invasion (OR: 3.69, *p* < 0.001), venous invasion (OR: 3.29, *p* = 0.005), collecting duct system invasion (OR: 4.91, *p* = 0.013), and necrosis (OR: 3.25, *p* < 0.001) are associated with low SRF of the ipsilateral kidney. The presence of a sarcomatous component was of borderline statistical significance (OR, 3.41; *p* = 0.059).

**Table 2:** Associated pathologic findings with decreased ipsilateral split renal function

	Univariate analysis		
Variables	OR	95% CI	*p*-value
Renal capsule invasion	1.53	1.11–2.12	0.010
Perirenal fat invasion	1.83	1.06–3.17	0.030
Renal sinus fat invasion	3.69	2.04–6.67	< 0.001
Sarcomatous component	3.41	0.95–12.2	0.059
Lymphatic invasion	3.07	0.73–13.0	0.127
Venous invasion	3.29	1.47–7.37	0.005
Collecting duct system invasion	4.91	1.40–17.2	0.013
Necrosis present	3.25	2.19–4.82	< 0.001

*OR* odds ratio, *CI* confidence interval

### Survival analysis predicting DFS

**Fig. 2:**
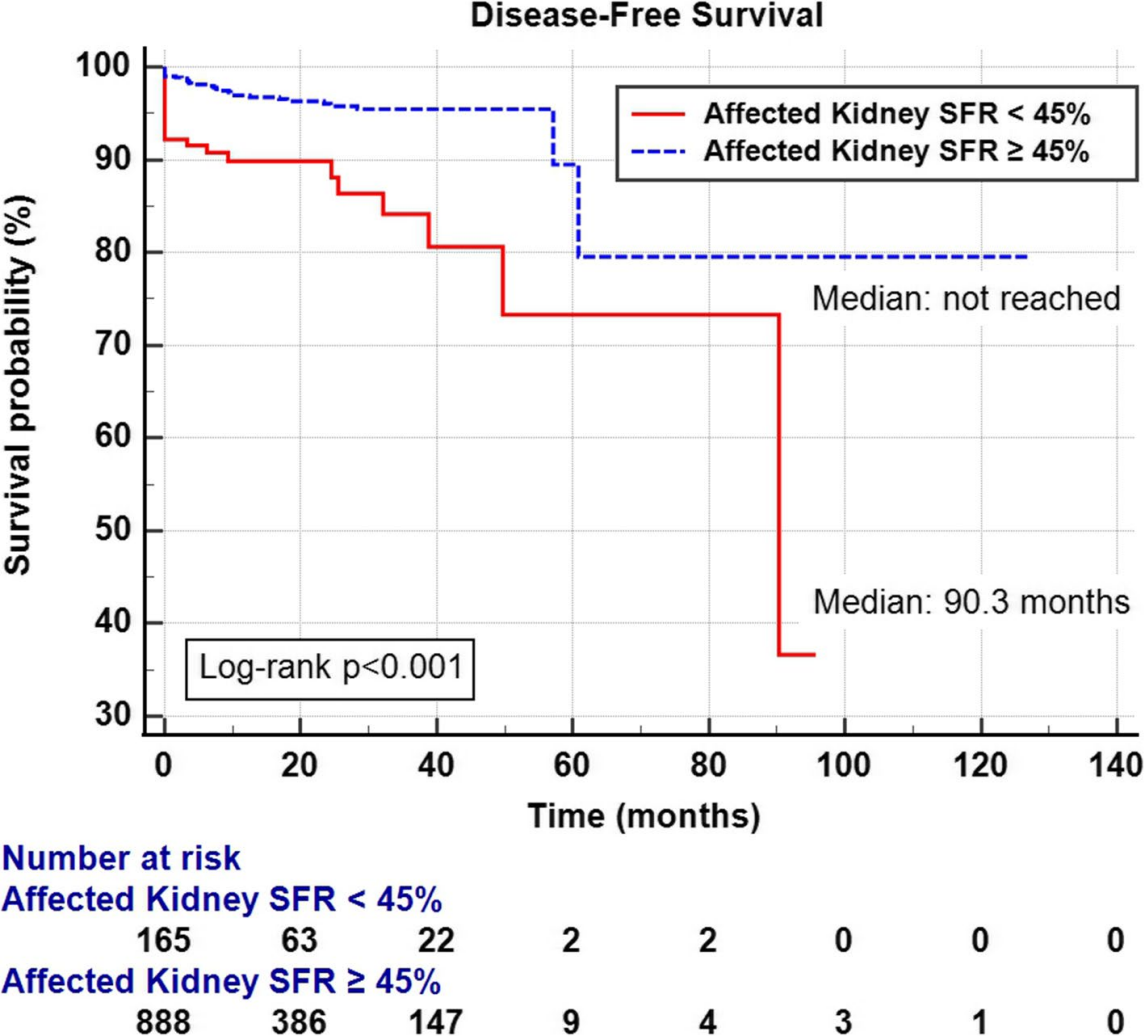
Kaplan–Meier curve of the effect of decreased ipsilateral SRF (< 45%) (red) maintained ipsilateral SRF (≥ 45%) (dotted blue) groups on disease-free survival

Univariate and multivariate Cox regression analyses were performed to determine factors affecting DFS. Univariate analysis identified age (> 60 years), higher SSIGN score and decreased ipsilateral SRF as significant risk factors (Table 3). In multivariate analysis, SSIGN (5–7) (hazard ratio [HR] 11.9, *p* < 0.001) and SSIGN (8–10) (HR 69.2, *p* < 0.001) were significantly associated with shortened DFS, while decreased ipsilateral SRF (HR 1.75, *p* = 0.065) showed borderline significance. Kaplan–Meier analysis was used to study the association between decreased ipsilateral SRF and DFS. The low ipsilateral SRF (< 45%) group showed shorter DFS than the other groups (median DFS: 90.3 months vs. not reached, *p* < 0.001) (Fig. 2).

**Table 3:** Univariate and multivariate analysis of risk factors for disease recurrence (Cox regression analysis)

	Univariate Cox Regression			Multivariate Cox Regression		
Variables	HR	95% CI	*p*-value	HR	95% CI	*p*-value
Age (> 60 years)	1.84	1.04–3.25	0.035	1.37	0.77–2.45	0.283
Sex (Male vs. Female)	1.93	0.97–3.83	0.061	1.32	0.66–2.66	0.437
Histology (clear cell vs non-clear cell)	1.54	0.66–3.62	0.318			
SSIGN score						
0–4	Reference			Reference		
5–7	14.0	7.36–26.5	< 0.001	10.59	5.29–21.2	< 0.001
8–13	77.9	38.2–159	< 0.001	59.2	27.8–126	< 0.001
DTPA SRF of affected kidney (< 45%)	3.94	2.29–6.77	< 0.001	1.75	0.97–3.18	0.065

*HR* hazards ratio, *CI* confidence interval, *DTPA* diethylenetriamine pentaacetic acid, *SRF* split renal function

### Survival analysis of the SSIGN subgroup in non-metastatic RCC

One thousand fifty-three patients of localized and locally advanced RCC were included for analysis. 977 patients (90.5%) showed SSIGN score 0–4, 84 patients (7.8%) with SSIGN score 5–7, and 18 patients (1.7%) with SSIGN score 8–13. Kaplan–Meier analysis was used to study the association between decreased ipsilateral SRF and DFS in SSIGN ≤ 4 and SSIGN > 4 subgroup, respectively. In SSIGN ≤ 4 (low risk of disease recurrence) group, the low ipsilateral SRF (< 45%) group showed significantly shorter DFS compared to the other group (log-rank *p* = 0.02), while there was no significant DFS difference in SSIGN > 4 subgroup (log-rank *p* = 0.134) (Supplementary Fig. 2).

## Discussion

When tumor cells colonize an organ and start proliferating, two types of growth patterns are observed. One is the type of tumor displacing the normal structure, causing mild compression damage to the surrounding tissue; in the other, the tumor invades or infiltrates the normal structure [14], leading to normal architecture disruption, followed by organ function loss. The latter type is associated with poor overall survival [14, 15]. Thus, efforts to define the molecular mechanism of invasive RCC and identify diagnostic biomarkers and therapeutic targets via a radio-pathological or radio-genomics approach have been made [16–18]. Herein, decreased ipsilateral SRF was associated with large tumor size, high T stage, and high Fuhrman grade, which are well-known prognostic factors. Moreover, most of the pathologically invasive features showed a significant association with ipsilateral renal function impairment, which supports our theory describing the causal relationship between invasive growth patterns and their impact on renal function deterioration. To examine its prognostic effect on DFS, we validated this novel risk factor using univariate and multivariate Cox regression analysis.

In addition to the direct destruction or compression effect mentioned above, several possible theories suggest that decreased ipsilateral kidney function could be associated with invasive and aggressive RCC. According to the competition theory of tumor growth [19], invasive growth phenotype acquisition dramatically alters the interaction with the surrounding tissue, rapidly destroying the normal tissue. The second is the dysregulated adjacent tissue theory. Genomic analysis revealed that the genetic features of normal adjacent tissues were different from those of normal tissues [20], which were enriched in pro-inflammatory signals and had dysregulated normal function. This theory is consistent with the desmoplasia phenomenon (connective tissue formation around the tumor stroma), which is aggravated in invasive cancers, resulting in excessive connective tissue accumulation [21]. Tumor-adjacent normal tissue undergoes functional damage; the degree of invasiveness aggravates the range of inflamed dysfunctional areas. This type of interaction between the tumor and surrounding normal tissue is clinically confirmed in several organs, including the breast [21], pancreas [22], and brain [14].

To assess the degree of tumor-induced organ dysfunction, we analyzed pre-operative Tc 99 m-DTPA renal scan data. In adult urology, Tc 99 m-DTPA is used to monitor postoperative renal function preservation/recovery after partial nephrectomy [23] or while deciding kidney donor suitability/laterality or monitoring postoperative compensatory hypertrophy [24]. We have accumulated data on pre-operative Tc 99 m-DTPA in patients who were planned to undergo partial or radical nephrectomy; thus, we decided to focus on SRF of the affected kidney. Decreased ipsilateral SRF seemingly represented tumor extension burden, while the overall estimated glomerular filtration rate (eGFR) did not show a significant difference between the maintained and decreased SRF groups (Table 1). Thus, the SRF factor, which is the relative ratio of unaffected to affected kidney function, is advantageous because it is not significantly affected by systemic or chronic kidney diseases that impact both kidneys. In this way, we could establish our novel hypothesis and discover that SRF is an important prognostic factor that correlates with the invasive behavior of kidney cancer. Despite the compensatory hypertrophy issues, increasing SRF of the contralateral kidney due to compensatory hypertrophy ultimately provides results in the same direction.

Decreased ipsilateral renal function on renogram prior to partial or radical nephrectomy has the following clinical implications. First, it may aid clinical decision-making for whom both partial and radical nephrectomy is being considered by stratifying both functional and oncological risk. In terms of functional aspect, decreased ipsilateral SRF may indicate low volume of the salvable functional renal parenchyme, thus there being little advantage of partial nephrectomy. Oncologically, since the risk of pathologic high T stage is higher in decreased ipsilateral SRF group (16.4% vs 8.9%, *p* = 0.003 in this study), this also supports the justification of radical nephrectomy. Second, we confirmed that the subgroup with a low SSIGN score (0–4), which is expected to have a lower chance of disease recurrence, was subdivided into two groups with significantly different prognosis by the ipsilateral SRF. Clinically, this indicates that even with low SSIGN scores, patients with decreased ipsilateral SRF should be more carefully followed up for the disease recurrence.

Collectively, in patients with RCC undergoing surgical resection, reduced ipsilateral renal function may represent aggressive behavior wherein the pathological association is verified. Previous studies have investigated RCC progression risk factors; however, robust evidence is unavailable. Although a prospective study is needed, our novel approach for analyzing the role of ipsilateral renal function may facilitate the risk stratification of patients with RCC, helping formulate a treatment strategy. Our novel marker is not significantly affected by systemic diseases because it measures differential renal function rather than objective renal function.

However, this study has several limitations. First, although this study had a relatively large population-based cohort, it was a retrospective study with innate limitations. To consolidate our results, a prospective randomized control study should be conducted to objectively compare the decreased and non-decreased ipsilateral renal function groups. Also, large-sized cohort validation should be followed to ascertain that decreased ipsilateral SRF is surely an independent predictive factor for the disease recurrence since we could not achieve statistical significance on multivariate Cox regression analysis. Second, due to the relatively short follow-up period, a small number of cancer-related deaths were noted; thus, the impact of ipsilateral renal function on overall survival was not assessed. Third, although it is pathologically proven to be associated with aggressiveness, we could not exclude the effect of compensatory hypertrophy in the contralateral kidney on SRF, which may be a confounding factor. Fourth, the association of imaging features of aggressive RCC on MDCT with decreased ipsilateral renal function on DTPA renal scan could not be assessed owing to the vast amount of data. Thus, our group is currently working on the artificial intelligence (AI)-based discrimination of invasive RCC. AI-based imaging feature extraction using MDCT in the lowered versus maintained SRF group would further provide insight into the novel radiologic features associated with poor prognosis in RCC.

## Conclusions

In patients with unilateral RCC, patients with lowered ipsilateral SRF showed a poor prognosis with pathologically invasive features. Although a prospective study is needed, our novel approach may facilitate the risk stratification of patients with RCC, helping formulate a treatment strategy.

## Supplementary Information

**Additional file 1:** **Supplementary Table 1. **Comparison of characteristics between maintained and decreased ipsilateral SRF in synchronous metastatic renal cell carcinoma.

**Additional file 2:** **Supplementary Fig. 1.** Inclusion and exclusion of patients in this study.

**Additional file 3:** **Supplementary Fig. 2.** Kaplan–Meier curve of the effect of decreased ipsilateral SRF (<45%) (red) and maintained ipsilateral SRF (≥45%) (dotted blue) groups in (**A**) SSIGN ≤4 and (**B**) SSIGN >4 group on disease-free survival in non-metastatic RCC.

## Data Availability

The datasets used and/or analysed during the current study are available from the corresponding author on reasonable request.

## Abbreviations

SRF: Split renal function
DFS: Disease-free survival
RCC: Renal cell carcinoma
MDCT: Multidetector computed tomography
DTPA: Diethylenetriamine pentaacetic acid
APCT: Abdomino-pelbic computed tomography
SSIGN: The stage, size, grade, and necrosis
OR: Odds ratio
HR: Hazard ratio
eGFR: Estimated glomerular filtration rate
