# Supplementary material for: Association between decreased ipsilateral renal function and aggressive behavior in renal cell carcinoma
Source: BMC Cancer. 2022 Nov 7;22:1143. doi: 10.1186/s12885-022-10268-1 (PMC9639309; doi:10.1186/s12885-022-10268-1)
Supplement: Supplementary file 1 — Additional file 1: Supplementary Table 1. Comparison of characteristics between maintained and decreased ipsilateral SRF in synchronous metastatic renal cell carcinoma. [file 12885_2022_10268_MOESM1_ESM.docx]

**Supplementary Table 1. Comparison of characteristics between maintained and decreased ipsilateral SRF in synchronous metastatic renal cell carcinoma**

|  | Maintained ipsilateral  SRF  (N=11) | Decreased ipsilateral SRF  (N=14) | *p*-value |
| --- | --- | --- | --- |
| Age (>60years) | 8 (88.9) | 7 (50.0) | 0.056 |
| Sex (n, %) |  |  | 0.964 |
| Male | 7 (77.8) | 11 (78.6) |  |
| Female | 2 (22.2) | 3 (21.4) |  |
| Renal sinus invasion | 1 (9.1) | 6 (46.2) | 0.047 |
| Perirenal invasion | 2 (18.2) | 5 (38.5) | 0.276 |
| Sarcomatous component | 0 (0.0) | 4 (30.8) | 0.044 |
| Necrosis present | 1 (12.5) | 9 (81.8) | 0.003 |
| T stage (n, %) |  |  | 0.066 |
| T1–T2 | 8 (72.7) | 5 (35.7) |  |
| T3–T4 | 3 (27.3) | 9 (64.3) |  |
| Fuhrman grade (n, %) |  |  | 0.017 |
| Grade 1–2 | 4 (36.4) | 0 (0.0) |  |
| Grade 3–4 | 7 (63.6) | 13 (100) |  |
| Pathology (n, %) |  |  | 0.366 |
| Clear cell | 11 (100) | 13 (92.9) |  |
| Non-clear cell | 0 (0.0) | 1 (7.1) |  |
| IMDC risk group |  |  | 0.102 |
| Intermediate risk | 11 (100) | 11 (78.6) |  |
| High risk | 0 (0.0) | 3 (21.4) |  |

SRF, split renal function; IMDC, International mRCC Database Consortium
