# Supplementary figures and images for: Association between decreased ipsilateral renal function and aggressive behavior in renal cell carcinoma
Source: BMC Cancer. 2022 Nov 7;22:1143. doi: 10.1186/s12885-022-10268-1 (PMC9639309; doi:10.1186/s12885-022-10268-1)

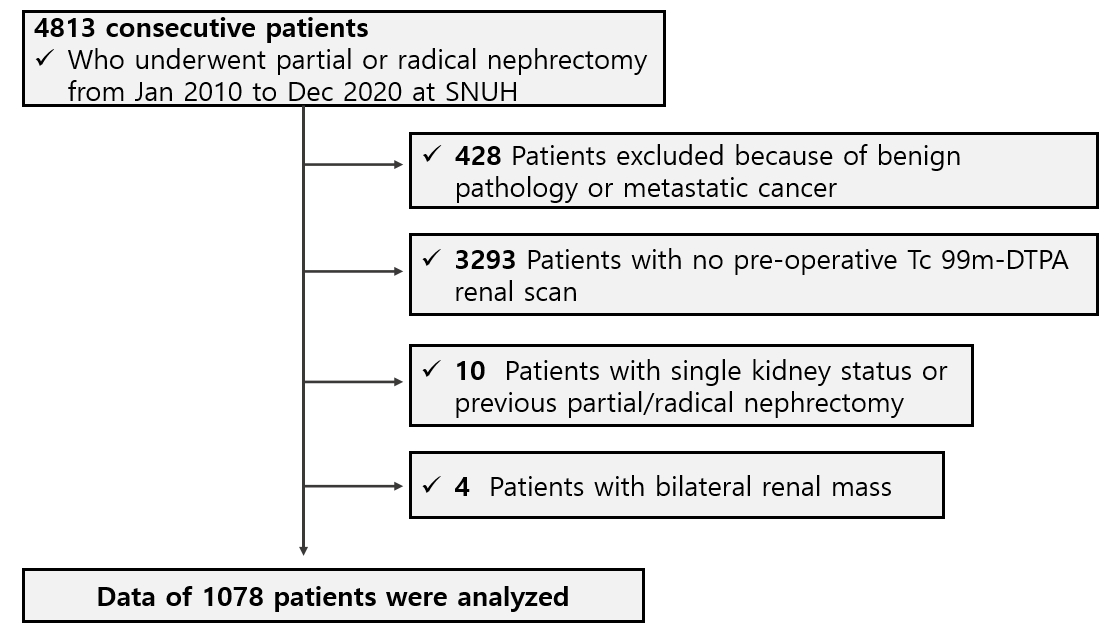

Supplement: Supplementary file 2 — Additional file 2: Supplementary Fig. 1. Inclusion and exclusion of patients in this study. [file 12885_2022_10268_MOESM2_ESM.jpg]

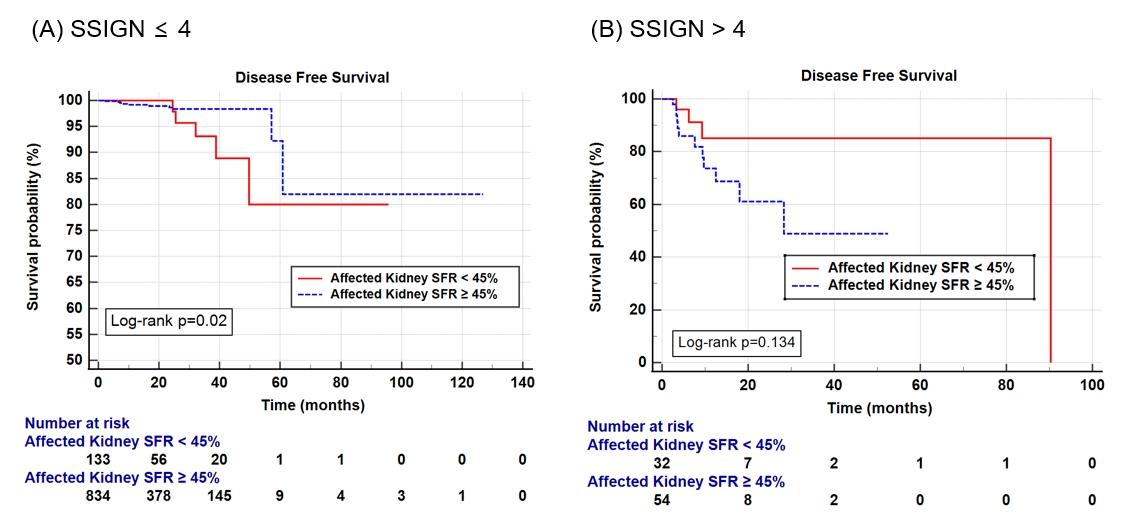

Supplement: Supplementary file 3 — Additional file 3: Supplementary Fig. 2. Kaplan–Meier curve of the effect of decreased ipsilateral SRF (<45%) (red) and maintained ipsilateral SRF (≥45%) (dotted blue) groups in (A) SSIGN ≤4 and (B) SSIGN >4 group on disease-free survival in non-metastatic RCC. [file 12885_2022_10268_MOESM3_ESM.jpg]
